# Molecular Detection of *Ehrlichia canis* in Dogs in Malaysia

**DOI:** 10.1371/journal.pntd.0001982

**Published:** 2013-01-03

**Authors:** Mojgan Nazari, Sue Yee Lim, Mahira Watanabe, Reuben S. K. Sharma, Nadzariah A. B. Y. Cheng, Malaika Watanabe

**Affiliations:** 1 Department of Veterinary Clinical Studies, Faculty of Veterinary Medicine, Universiti Putra Malaysia, Selangor, Malaysia; 2 Department of Veterinary Pathology and Microbiology, Faculty of Veterinary Medicine, Universiti Putra Malaysia, Selangor, Malaysia; University of Texas Medical Branch, United States of America

## Abstract

An epidemiological study of *Ehrlichia canis* infection in dogs in Peninsular Malaysia was carried out using molecular detection techniques. A total of 500 canine blood samples were collected from veterinary clinics and dog shelters. Molecular screening by polymerase chain reaction (PCR) was performed using genus-specific primers followed by PCR using *E. canis* species-specific primers. Ten out of 500 dogs were positive for *E. canis*. A phylogenetic analysis of the *E. canis* Malaysia strain showed that it was grouped tightly with other *E. canis* strains from different geographic regions. The present study revealed for the first time, the presence of genetically confirmed *E. canis* with a prevalence rate of 2.0% in naturally infected dogs in Malaysia.

## Author Summary

Canine vector-borne diseases are a worldwide concern particularly in the tropics and sub-tropics that provide favourable climatic conditions for the vectors. Malaysia, a tropical paradise, is thus home to a wide range of vectors as well as the pathogens that they harbor. *Ehrlichia canis*, a ubiquitous tick-borne pathogen of dogs, is the causative agent of canine monocytic ehrlichiosis, the most common clinically significant tick-borne disease of dogs in Malaysia. The pet explosion coupled with the increasing number of stray dogs, has resulted in a surge in vector-borne diseases in companion animals in Southeast Asia. Despite this, there is very little published information regarding this subject in Malaysia. There are only two published studies on *E.canis* in Peninsular Malaysia based on traditional light microscopic detection and antibody detection techniques. This disease has been notoriously difficult to diagnose based on the traditional methods. This research investigates this important disease of canids using molecular techniques for the first time in Malaysia providing a more accurate picture of its presence and prevalence in the country.

## Introduction

*Ehrlichia canis* is a gram-negative obligatory intracellular bacterium with a tropism for monocytes and macrophages in the family *Anaplasmataceae* and order *Rickettsiales* [1], [2]. Canine monocytic ehrlichiosis (CME) caused by *E. canis* is a tick-borne disease of dogs. *E. canis* is transmitted by the brown dog tick *Rhipicephalus sanguineus* [3], [4]. The disease was first described in 1935 in Algeria, as a febrile sickness associated with leukopenia, thrombocytopenia, depression and anemia in several dogs [1]. Some closely related pathogens, including *Ehrlichia ewingii*, *Ehrlichia chaffeensis*, *Anaplasma phagocytophilum* and *Neorickettsia risticii*, are shown to cause similar clinical and hematological manifestations in dogs as well [2], [5]. However, *E. canis* is responsible for the most common and clinically severe form of canine ehrlichiosis, and may also be a cause of human ehrlichiosis [6], [7].

Because rickettsiales are able to infect a broad range of hosts, and multiple pathogens can co-exist in both vertebrate and invertebrate hosts, the availability of a rapid, highly sensitive, and specific test that can diagnose one or more pathogens, including co-infections, in a test sample will be valuable for timely diagnosis and treatment [8], [9]. Traditional diagnostic techniques including hematology, cytology, serology and isolation are valuable diagnostic tools for CME, however it is believed that molecular techniques make the most appropriate means of diagnosis of *E. canis* infection, and would be useful for monitoring and controlling the spread of infection from ticks [10]. Moreover, a multiplex molecular test would be a valuable tool in studies to evaluate the impact of co-infections on the disease outcome, as well as in studies to assess vaccines and therapeutics [9]. Microscopic visualization of morulae in peripheral blood leukocytes may be the simplest test, but it is also the least sensitive technique. Currently serological tests are the most commonly practiced method for diagnosis of *E. canis* infection. These serological tests reflect the quantity of antibodies present in the serum and therefore indicate exposure but not the severity of disease and the duration of infection [11]. Furthermore, antibodies are usually absent during the first two weeks of onset [11]. Additionally antibodies against several other ehrlichial organisms might cross-react with *E. canis* and complicate the serological diagnosis [12]. False negative results are another common feature of serological tests and may occur due to the early stage of the disease and lack of antibody which may further impact the final diagnosis [13].

Conversely, polymerase chain reaction (PCR) is a sensitive method of detection of acute monocytic ehrlichiosis in dogs; in fact it is designed to aim for the organism itself which makes PCR an invaluable technique capable of detecting traces of pathogen even before the onset of clinical signs [14]. Therefore, the advantages of molecular detection of *Ehrlichia* include diagnosis before the development of antibodies in early stages of disease and identifying new species and also closely related species of *Ehrlichia* using species-specific primers and sequencing [15].

To date the presence of ehrlichial agents in dogs in Malaysia has not been investigated using molecular techniques and therefore, this study was undertaken to detect *E. canis* DNA and to determine the prevalence of the disease caused by this pathogen in dogs in Malaysia.

## Materials and Methods

### Ethics statement

The research was conducted as per the guidelines of the Animal Care and Use Committee, Faculty of Veterinary Medicine, Universiti Putra Malaysia. This committee follows the Australian code of practice for the care and use of animals for scientific purposes. The committee did not deem it necessary for this research group to obtain formal approval to conduct this study.

A total of 500 blood samples were collected from dogs in Peninsular Malaysia, comprising 177 samples from stray dogs at shelters around Selangor state (144) and Langkawi Island (33), and 323 samples from dogs that were presented to private veterinary clinics from Selangor (86) Johor (30), Melaka (27), Sabah (3), and the veterinary teaching hospital at Universiti Putra Malaysia (UPM), Selangor (177) for routine health care or specific treatment. Samples were collected in EDTA-anticoagulant tubes once a week randomly from February 2009 to February 2010, and stored at −20°C until further use. Information (age, breed, sex, and all laboratory results) of all the clinic cases was recorded, and the sex of the stray dogs was noted (Table 1).

**Table 1:** Summary of prevalence of *E.canis* among clinic cases based on sex, breed and age.

Criteria	Total number of dogs	No of *E. canis* positives (%)
**Sex Male**	171	4 (2.3)
**Female**	152	0 (0.0)
**Breed Pure-breed**	168	0 (0.0)
**Cross-breed**	155	4 (2.6)
**Age (years) 0–3**	119	2 (1.7)
**3–6**	84	1 (1.2)
**6–9**	76	1 (1.1)
**9–12**	34	0 (0.0)
**12–15**	10	0 (0.0)
**Total**	323	4 (1.2)

### DNA extraction

DNA was extracted from whole blood (200 µl) following the QIAamp animal blood and Tissue Kit procedure (QIAGEN GmbH, Hilden, Germany), adjusted in 200 µl of Tris- EDTA (TE) buffer and stored at −20°C until further use.

### PCR amplification

Standard screening conventional PCR was performed on all 500 samples using genus-specific primers; forward EHR16SD (5′- GGTACCYACAGAAGAAGTCC-3′) and reverse EHR16SR (5′-TAGCACTCATCGTTTACAGC-3′) [16]. Second PCR was performed on positive samples in the screening PCR using the *E. canis* species-specific set of primers; forward CANIS (5′-CAA-TTA-TTT-ATA-GCC-TCT-GGC-TAT-AGG-A-3′) and reverse GA1UR (5′-GAG-TTT-GCC-GGG-ACT-TCT-TCT-3)′ that amplifies approximately a 409 bp fragment of the 16S rRNA gene of *E. canis* [17], [18]. After detecting positive samples, in order to amplify a longer fragment of the 16SrRNA gene of *E. canis* including the divergent region, another PCR was performed with oligonucleotide primers: FD1 (5′-AGA-GTT-TGA-TCC-TGG-CTC-AG-3′) and Rp2 (5′-ACG-GCT-ACC-TTG-TTA-CGA-CTT-3′) [19]. The PCR amplification was set up within a 25 µl reaction mixture containing 5 µl of DNA template and 20 µl of master mix (2.5 µl 10× buffer without MgCl_2_, 10 µM of dNTP, 5 mM MgCl_2_, 0.8 µM of each primer, 5 units of Taq polymerase, and sterile distilled water to a final volume of 20 µl). The thermal cycling procedure was; 1 cycle of 5 minutes at 95°C, 40 cycles of 30 seconds at 95°C, 30 seconds at 62°C, 60°C or 63°C depending on the primers used, 1.30 minutes at 72°C, and final cycle of 5 minutes at 72°C. Sterile distilled water and DNA of an *E. canis* positive dog were included as a negative and positive control, respectively.

The amplification products were visualized on a 1.5% agarose gel after electrophoretic migration for 40 minutes at 100 voltages. The gels were stained with ethidium bromide for 10 minutes and visualized by UV illumination.

### Sequence and similarity analysis

Amplicons were extracted using the QIAPCR purification kit (QIAGEN) for direct sequence analysis using ABI prismTM BigdyeTM terminator cycle sequencing Ready reaction kit V.3.1. All sequences were aligned manually using ClustalW program (www.ebi.ac.uk/clustalw). For comparing and analyzing the nucleotide sequences the BLAST program (http://www.ncbi.nlm.nih.gov/BLAST) was used. A similarity tree was inferred using the neighbor- joining method, MEGA software version 5.

### Statistical analysis

The statistical analysis was performed using the chi-square test and the Fisher exact test to determine the relation between the observed variables; prevalence between the stray dogs and clinic cases, sex of the animals (male and female), age, clinical signs (symptomatic or asymptomatic) and the dispersion of these frequencies.

## Results

**Figure 1:**
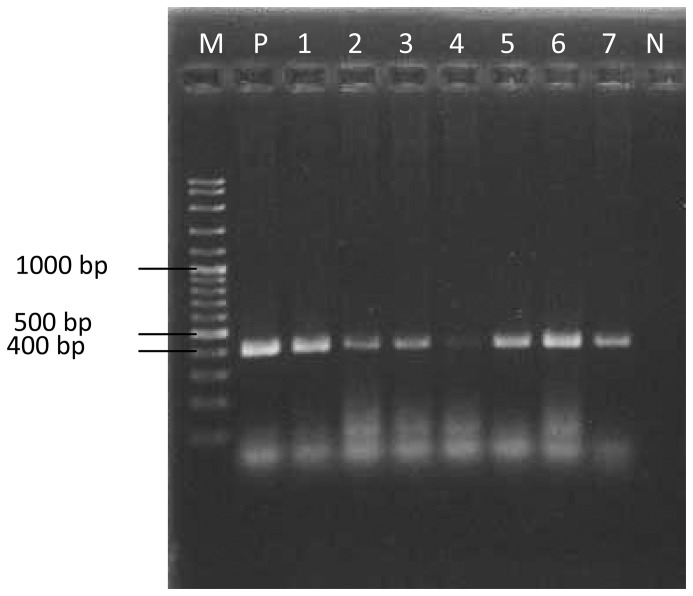
1.5% agarose gel stained with ethidium bromide. Amplification of the 16S rRNA gene with CANIS/GA1UR primers, approximately 409 bp, lane M = 100 bp DNA ladder, lane P = positive control, lane 1–7 = positive samples, lane N = negative control.

Five hundred blood samples (323 clinic cases and 177 stray dogs) were evaluated using PCR in this study out of which ten were identified as *E. canis* positive, giving an overall prevalence rate of two (2.0%) percent. The prevalence of E. canis was calculated as 1.2% (4 of 323) amongst the clinic group, and 3.4% (6 of 177) among the stray dogs. There was no significant difference in the prevalence of *E. canis* between stray dogs and clinic cases (*X^2^* = 0.400, *P* = 0.527). The amplification with species-specific primers CANIS/GA1UR produced a clear single band of approximately 409 bp (Figure 1). The ten positive PCR products were purified and sequenced. BLAST analysis confirmed the isolation of *E. canis* with 100% identity to other registered *E. canis* strains in GenBank. The nucleotide sequence was deposited in NCBI GenBank database (accession number JF429693.1). This is the first confirmed detection of *E. canis* DNA from dogs in Malaysia.

Among the clinic cases 27 (8.3%) dogs had both thrombocytopenia and anemia, but only one of them was positive for *E. canis*. However thrombocytopenia appeared to be more consistently associated with the disease as three out of four positive dogs had thrombocytopenia and this proved to be of statistical significance (*P* = 0.017).

The major part of the 16S rRNA sequences (1384 bp) using primers FD1/Rp2, amplified from the *E. canis* positive dogs, was 100% identical with the corresponding sequences from *E. canis* strains in different geographical areas of the world. Nucleotide differences in 16S rRNA sequences among *E. canis* strains from different geographical areas showed very few differences. An *E. canis* similarity tree was inferred using the neighbor- joining method, MEGA software version 5. A similarity tree of *E. canis* Malaysia based on nucleotide sequences showed that the Malaysia strain was grouped tightly with other *E. canis* strains from different geographic regions (accession numbers EU263991.1, EU106856.1, AF373613.1).

## Discussion

In the present study *E. canis* was successfully amplified using molecular techniques and this represents the first molecular survey of this pathogen in Malaysia. The only published investigation based on detection of *E. canis* via light microscopic examination of peripheral blood films was carried out over 25 years ago revealing a prevalence rate of only 0.2% in dogs, and recently the prevalence of *E. canis* infection was determined to be 15% in Perak state of Malaysia using indirect immunofluorescence assay (IFA) [20], [21].

Due to limitations of light microscopic examination for the detection of *E. canis*, it was imperative to study the prevalence using more reliable diagnostic methods. Furthermore, due to high prevalence rates of even up to 30% around the world, and because *Ehrlichia* species are the etiological agents of emerging and life-threatening tick-borne disease in domestic animals, there was a pressing need to determine actual prevalence rates in Malaysia [22]–[24].

In the current study, a relatively large number of blood samples from both stray dogs and clinic cases were subjected to PCR, a rapid, highly sensitive, and specific method for the detection of *E. canis*. The study revealed that the molecular prevalence of *E. canis* in the tested samples was 2.0%. This low prevalence rate is interesting, as one would expect Malaysia to be a highly endemic region for *E. canis* due to the suitable climate and the abundance of the tick vector. Thus further studies are needed to detect *E. canis* DNA from ticks, as there would be no transmission to dogs if the ticks are not infected.

Prevalence rates were low in both clinic cases and stray dogs. The reasons for the low detectable rates requires further investigation however it is important to bear in mind that subclinical and chronic ehrlichial infections are not as readily diagnosed as acute infections when canine blood is used for the detection of *E. canis*. Therefore, ideally, PCR using both blood and splenic aspirates should be considered to overcome this limitation [25]. Furthermore PCR sensitivity varies between laboratories and this fact may have contributed to the low number of positive dogs identified.

Analysis of laboratory findings of clinic dogs revealed few associations between hematological findings and *E. canis* infection status (Table 2). Furthermore, although 5 (1.5%) dogs had laboratory findings typical of CME: thrombocytopenia, anemia, and lymphopenia, none were positive for *E. canis*. Among the clinic cases 27 (8.3%) dogs had both thrombocytopenia and anemia, but only one of them was positive for *E. canis*. These results however highlighted an important point; over diagnosis or misdiagnosis may result if a diagnosis is made solely on clinical or hematological findings as they are not specific for CME. At the same time it may also have been possible that some of these dogs were subclinically or chronically infected and thus had lab findings consistent with CME but PCR was unable to detect the organism. Therefore a reliable diagnosis of *E. canis* can only be made based on a combination of clinical signs, laboratory test results, serological tests, and molecular methods of detection.

**Table 2:** Association between *E.canis* infection status and abnormal laboratory findings for clinic cases.

Lab findings	No of test positives/No tested (%)	No of *E. canis* positives/No of test positives (%)	Normal values
Thrombocytopenia	55/323 (17%)	3/55 (5.4%)	200–500×10^9^/L
Thrombocytosis	8/323 (2.5%)	0/8 (0%)	200–500×10^9^/L
Anemia	65/323 (20.1%)	1/65 (1.5%)	0.35–0.55 L/L
Hypoalbuminemia	47/323 (14.6%)	2/47 (4.2%)	25–40 g/L
Hyperalbuminemia	37/323 (11.5%)	0/37 (0%)	25–40 g/L
Lymphopenia	36/323 (11.1%)	0/36 (0%)	1.5–4.8×10^9^/L
Lymphocytosis	5/323 (1.5%)	0/5 (0%)	1.5–4.8×10^9^/L
Hyperglobulinemia	84/323 (26.2%)	2/84 (2.3%)	25–45 g/L
Hypoglobulinemia	19/323 (5.9%)	0/19 (0%)	25–45 g/L
Neutrophilia	61/323 (18.9%)	0/61 (0%)	3–11.5×10^9^/L

As this was the first molecular study of *E. canis* it was imperative to carry out genetic characterization of the Malaysian strain for a better understanding of the pathogen in Malaysia. All 1384 base pair (bp) amplified sequences of the *E. canis* 16S rRNA of the Malaysia strain were found to be identical to other deposited strains in NCBI GenBank. Nucleotide differences in 16S rRNA sequences among *E. canis* strains from different geographical areas showed very few differences which could be explained by the fact that the genetic profile of canine *E. canis* strains based on the 16S rRNA gene is highly conserved.

In conclusion, *E. canis* DNA was detected for the first time from dogs in Malaysia and the overall prevalence rate of *E. canis* in naturally infected dogs was 2.0%. The detection of *E. canis* DNA via PCR in this study confirms the presence of the infection in both the pet and stray dog populations in Malaysia.
